# Isobavachalcone From *Cullen corylifolium* Presents Significant Antibacterial Activity Against *Clostridium difficile* Through Disruption of the Cell Membrane

**DOI:** 10.3389/fphar.2022.914188

**Published:** 2022-07-22

**Authors:** Xi-Wang Liu, Ya-Jun Yang, Zhe Qin, Shi-Hong Li, Li-Xia Bai, Wen-Bo Ge, Jian-Yong Li

**Affiliations:** Key Laboratory of New Animal Drug Project of Gansu Province, Key Laboratory of Veterinary Pharmaceutical Development, Ministry of Agriculture and Rural Affairs, Lanzhou Institute of Husbandry and Pharmaceutical Sciences of CAAS, Lanzhou, China

**Keywords:** *Clostridium difficile*, psoraleae fructus, antibacterial activity, isobavachalcone, cell membrane integrity

## Abstract

**Background:** *Clostridium difficile* infection (CDI) has been widely reported in human and animals around the world over the past few decades. The high relapse rate and increasing drug resistance of CDI make the discovery of new agents against *C. difficile* fairly urgent. This study aims to investigate the antibacterial activity against *C. difficile* from traditional Chinese herb medicine *Cullen corylifolium* and confirm its active components.

**Methods:** Phenolic extract from the seeds of *C. corylifolium* was prepared routinely and the contents of relative flavonoids were determined by High Performance Liquid Chromatography (HPLC). *In vitro* antibacterial activities of the phenolic extract and its major components were tested. The influence of the major components on cell membrane was investigated with membrane integrity by SEM and propidium iodid uptake assay. Cytotoxicity of the extract and its active compounds on Caco-2 cell line was assessed by CCK-8 kit. The *in vivo* therapeutic efficacy of IBCL was evaluated on the mice model.

**Results:** Phenolic extract was found to be active against *C. difficile* with minimum inhibitory concentrations (MIC) of 8 μg/mL. As the major component of the extract, IBCL was the most active compound against *C. difficile*. The MIC of IBCL and 4MBCL were 4 μg/ml and 4 μg/ml, respectively. Meanwhile, PFPE, IBCL, and 4MBCL showed rapid bactericidal effect against *C. difficile* in 1 h, which was significant compared to antibiotic vancomycin. Mechanism studies revealed that IBCL can disrupt the integrity of the cell membrane, which may lead to the death of bacteria. PFPE was low cytotoxic against Caco-2 cells, and the cytotoxicity of IBCL and 4MBCL were moderate. Symptoms of CDI were effectively alleviated by IBCL on the mice model and weight loss was reduced. From death rates, IBCL showed better efficacy compared to vancomycin at 50 mg/kg dosage.

**Conclusion:** As the major component of phenolic extract of *C. corylifolium* seeds, IBCL showed significant antibacterial activity against *C. difficile in vitro* and rapidly killed the bacteria by disrupting the integrity of the cell membrane. IBCL can significantly prevent weight loss and reduce death caused by CDI on the mice model. Therefore, IBCL may be a promising lead compound or drug candidate for CDI.

## Introduction

*Clostridium difficile* (*C. difficile*) is a Gram-positive, anaerobic, spore-forming, and toxin-producing *bacillus* that was first separated from infants in 1935 (Hall and O'Toole, 1935). Over the past two decades, increasing studies have found that *C. difficile* is the most frequently reported nosocomial pathogen, and it causes a serious intestinal infection that is named *C. difficile* infection (CDI). CDI can be widely transmitted by spores of *C. difficile*, which are resistant to heat, acid, and antibiotics. The spores are plentiful in healthcare facilities and are found in low levels in the environment and food supply, allowing for both nosocomial and community transmission. The US Centers for Disease Control and Prevention estimated that almost 500,000 patients suffered CDI, with 29,000 attributable deaths in the United States in 2011 (Lessa et al., 2015). In Europe, about 124,000 patients developed healthcare-associated CDI each year between 2011 and 2012 (Smits et al., 2016). Currently, antibiotics are the mainstays of CDI therapy, including metronidazole, vancomycin, and fidaxomicin. However, the high recurrence of CDI (20–60%) from the side effect of antibiotics to normal intestinal flora causes the treatment of recurrent CDI to be more complicated (Leffler and Lamont, 2015). Furthermore, antibiotic resistance has continued to emerge following the extensive and widespread use of antibiotics (Peng et al., 2017). Consequently, the discovery of new drugs is becoming increasingly urgent.

**FIGURE 1:**
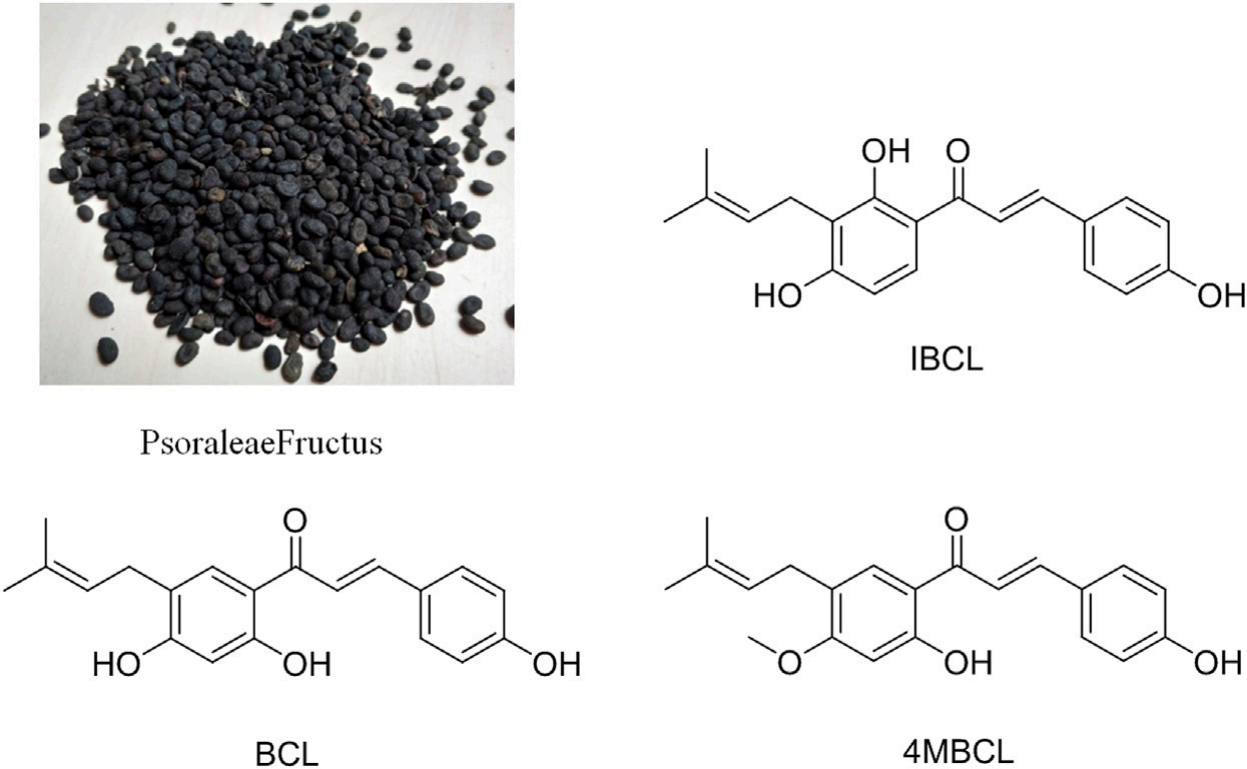
Photograph of Psoraleae Fructus and structures of IBCL, BCL, and 4MBCL.

As the seeds of *Cullen corylifolium*, Psoraleae Fructus (PF, Figure 1) is a traditional Chinese medicine (TCM) that has been used for thousands of years and is officially listed in China Pharmacopoeia. PF can effectively warm the kidneys and the whole body and can also control diarrhea (Chinese-Pharmacopoeia-Commission, 2015). Recent studies have shown that the extract and related components of PF exhibited various biological activities, such as antibacterial activity (Cui et al., 2015), insecticidal activity (Dua et al., 2013), antioxidant activity (Xiao et al., 2010), and anti-tumor activity (Wang et al., 2016). Phytochemical studies have indicated that flavonoids, coumarins, and meroterpenes are the main active components of PF (Zhang et al., 2016).

In this paper, we investigate the *in vitro* antibacterial activity of PF extract against *C. difficile* and confirm the active compounds in the extract. The phenolic extract of PF (PFPE), isobavachalcone (IBCL, Figure 1), and 4′-O-methylbavachalcone (4MBCL, Figure 1) have shown significant antibacterial activity against *C. difficile* and have rapidly killed *C. difficile* in a concentration-dependent manner. Further studies have found that the cell membrane of *C. difficile* was quickly destroyed by IBCL, 4MBCL, and PFPE. Cytotoxicity assay has revealed that PFPE has low cytotoxicity, while IBCL and 4MBCL are moderately cytotoxic. IBCL can be a significant protection for CDI in the mice model.

## Materials and Methods

### Compounds and Reagents

The PF was purchased from Huanghe TCM market in Lanzhou city (Gansu province) in Northwest China. PF was authenticated by Prof. Zhi-Gang Ma of Pharmacy College of Lanzhou University. Psoralen (PL), Isopsoralen (IPL), Bavachin (BVC), Neobavaisoflavone (NBIF), Bavachinin (BVCN), Corylin (CRL), Psoralidin (PLD), Bavachalcone (BCL), IBCL and 4MBCL, gentamycin, and clindamycin were obtained from YuanYe Bio-Technology Co., Ltd. (Shanghai, China) and the purities were above 98%. Vancomycin, metronidazole, Colistin, Kanamycin, and the cell viability kit were purchased from Sigma-Aldrich. The strains of *C. difficil*e ATCC 43255 and BAA-1803 were obtained from American Type Culture Collection (ATCC, Manassas, VA, United States). *C. difficile* strain CICC 22951 was purchased from China Center of Industrial Culture Collection (CICC, Beijing, China). Caco-2 cell was purchased from Shanghai Cell Bank of Chinese Academy of Sciences (Shanghai, China)). Brain heart infusion (BHI) broth and agar were supplied from Guangdong Huankai Microbial Technology Co., LTD (Guangdong, China). Unless otherwise stated, all *C. difficile* strains were routinely grown in BHI broth or agar at 37°C in anaerobic conditions (85% N_2_, 10% H_2_, 5% CO_2_) in a Forma Anaerobic System (Thermo Scientific™, United States). Stock solutions of all compounds were prepared in DMSO and stored at −20°C.

### Preparation of Phenolic Extract of PF (PFPE)

The dried PF was ground into powder (24 mesh, 500 g) and extracted twice with 75% ethanol (2.5 L) for 12 h at room temperature with continuous stirring (300 rpm/min). The desired solution was combined and evaporated under vacuum (50°C, 150 mbar) to a volume of 0.5 L. Liposoluble components were twice removed by n-hexane (200 ml), and the residual suspension was alkalified with 5% Na_2_CO_3_ solution (150 ml) to pH 10. The insoluble substance was removed by Buchner funnel. The following filtrate (600 ml) was acidized under stirring (300 rpm/min) with 1 mol/L HCl to pH 3–4. The produced precipitates were obtained and dried to give the desired PFPE (5.6 g).

### HPLC Analysis

The Agilent 1290 High Performance Liquid Chromatography (HPLC) system (Agilent, United States) was used with a DAD detector at 254 nm. The column was Agilent Poroshell 120 EC C18 (150 mm × 4.6 mm, 2.7 μm), and the column temperature was 35°C. The gradient mobile phase consisted of 0.5% phosphoric acid water (A) and methanol (B). The ratio of A: B was as followed: 0 min, 60∶40; 20 min, 0∶100; 25 min, 0∶100. The flow rate was 0.8 ml/min, and the injection volume was 5 μl.

Individual and mixed stock solutions (1 mg/ml) of PL, IPL, BVC, NBIF, BVCN, CRL, PLD, BCL, IBCL, and 4MBCL were prepared in methanol and serially diluted to concentrations of 200, 100, 50, 25, 12.5, 6.25, 3.12, and 1.56 μg/ml. PFPE was dissolved in 50% methanol (1 mg/ml) and filtered in 0.22 μm syringe filter.

For the content determination of PF, about 0.5 g powder (24 mesh) of PF was ultrasonically extracted with 50 ml 85% methanol solution in a flask for 30 min at room temperature. Subsequently, the loss of volume was compensated by 85% methanol and finally filtered through 0.22 μm filter before detection on HPLC (Zhang et al., 2017).

Peaks were identified by comparing their retention time with the reference compounds. Concentrations of detected samples were quantitatively calculated using the corresponding curves of the reference compounds as standards.

### Determination of MIC

The MICs of PFPE, compounds and control antibiotics were tested on 96-well microplate by broth microdilution method (Jorgensen et al., 2015). Briefly, *C. difficile* (ATCC 43255, BAA-1803, and CICC 22951) from a single colony was cultured in BHI broth to logarithmic phase (OD_600_ = 0.5–0.6) and diluted 1∶10 in BHI to approximately 1 × 10^6^ CFU/ml within 15 min; 10 μl stock solutions of PFPE, compounds, and control antibiotics were added to 190 μl BHI broth and two-fold serially diluted on a 96-well microplate. Then, 100 μl of the prepared bacteria culture was inoculated to each well to a desired bacteria concentration of 5 × 10^5^ CFU/ml. The final concentrations of compounds ranged from 64 μg/ml to 0.06 μg/ml. Solvent control (DMSO) and blank control (BHI) were also set. After anaerobically incubation at 37°C for 24 h, the lowest concentration of compounds that inhibited visible growth was recorded as the MIC. The MICs were also ascertained by agar incorporation on BHI agar to confirm the activities seen in broth. Essentially, cultures in BHI broth were multipoint inoculated onto various BHI agar plates with drugs (0.25–32 μg/ml) and incubated for up to 48 h before recording the MICs (Hurdle et al., 2011).

### Determination of Minimum Bactericidal Concentration

MBCs of PFPE, active components, and antibiotics against stationary-phase *C. difficile* were tested on 24-well plates as described in Wu et al. (2014). Briefly, 900 μl of stationary-phase (OD_600_ nm > 1.2) culture was added to 100 μl of two-fold serial dilution of compounds in phosphate-buffered saline (PBS). Following 48 h incubation, viable cells were enumerated on BHI agar. MBCs were recorded as the lowest concentration of killing ≥3 Logs of starting cells. All MBCs were performed at least twice.

### Growth Curves of C. difficile

*C. difficile* ATCC 43255 was cultured in 5 ml BHI broth for approximately 16 h. The overnight culture was diluted 1∶100 in BHI medium to approximately 1 × 10^6^ CFU/ml. Different concentrations of IBCL, 4MBCL, and PFPE in BHI medium were added into a 96-well microplate and mixed with an equal volume of bacterial dilutions. The microplates were incubated anaerobically at 37°C. The growth curves were established according to the absorbance of OD_600_ nm with an interval of 2 h by Multiskan™ microplate reader (Thermo Scientific). All of the measurements were done in triplicates for absorbance determination (Karaman et al., 2017).

### Time Kill Studies

Culture of *C. difficile* ATCC 43255 was prepared by inoculating fresh BHI broth with a single colony. Following overnight (18–20 h) incubation, the culture reached to a desired concentration of ∼10^8^ CFU/ml (OD_600_ nm > 1.2). Different volumes of stock solution of PFPE, IBCL, 4MBCL, and PFPE were added to a desired concentration of 2 × MIC and cultured anaerobically at 37°C. Meanwhile, 100 μl aliquots were sampled after incubation for 0, 15, 30, 45, and 60 min. Next, bacteria solutions were tenfold serially diluted and plated on BHI agar to calculate the colony-forming units (CFUs) after incubation for 48 h. The experiments were performed in two independent replicates.

### Scanning Electron Microscopy

*C. difficile* ATCC 43255 was cultured in BHI broth to logarithmic phase (OD_600_ = 0.5–0.6). Proper volume stock solutions of PFPE, IBCL, and 4MBCL were added to achieve 2 × MIC concentration (PFPE = 16 μg/ml, IBCL = 8 μg/ml, 4MBCL = 8 μg/ml) with DMSO as a positive control. After incubation for 2 h, the medium was removed by centrifugation at 4000 *g* for 5 min. The precipitates were washed by PBS for three times and fixed with 2.5% glutaraldehyde in PBS at 30°C for at least 12 h. Samples were stored at 4°C before further processing. The fixed bacteria were subjected to dehydration processes via incremental percentages of ethanol for 10 min each (50, 75%, 95%, and 100% ethanol) before being air-dried. The coverslips were then immersed in the hexamethyldisilazane sputtered in gold and subsequently examined under the SEM (HITACHI Regulus 8,100, Japan).

### Propidium Iodide Uptake

A PI uptake assay was performed to confirm whether the antibacterial activity of IBCL was due to the damage of membrane and the consequent leakage of intracellular contents. Briefly, stationary-phase cells in BHI were exposed to different concentrations of IBCL and incubated anaerobically at 37°C. Samples (1 ml) were obtained after 1, 2, 4, and 6 h and centrifuged at 4000 *g* for 10 min. The resulting cell pellets were resuspended in 1 ml PBS containing 5 μmol/L of PI and dyed anaerobically for 10 min. The samples were then centrifuged and twice washed with PBS to remove the excess dye. Finally, 100 μl of the desired cells in PBS solution were transformed to flat bottom 96-well plates. PI fluorescence was measured in a plate reader (PE, Enspire) at excitation 544 nm and emission of 620 nm.

### Cell Viability Assay

Caco-2 cell viability was tested using the Cell Counting Kit-8 (CCK-8) (He et al., 2018). Briefly, 1 × 10^5^ cells were seeded in 96-well plates and incubated for 24 h at 5% CO_2_, 37°C. Meanwhile, 100 μl medium containing PFPE, IBCL, and 4MBCL were added to cell plates with DMSO less than 1% (*v/v*). After incubation for 12 h, 10 μl CCK-8 reagent was added to each well. Cells were incubated for 2 h at 37°C in a humidified 5% CO_2_ atmosphere. Finally, cell viability was recorded by measuring the absorbance at 450 nm using a microplate reader (Multiskan™ FC; Thermo Scientific™, United States).

### Animal Model

All of the procedures were conducted in accordance with an approved protocol from the Institutional Animal Care and Use Committee at the Lanzhou Institute of Husbandry and Pharmaceutical Sciences of CAAS. Balb/C mice (approximately 20 g) were orally administered 10^5^ CFU of *C. difficile* (ATCC 43255) strain after receiving antibiotic treatment in the drinking water for 3 days. The antibiotic mixture contains: kanamycin (0.4 mg/ml), gentamicin (0.035 mg/ml), conlistin (850 U/ml), metronidazole (0.215 mg/ml) and vancomycin (0.045 mg/ml). The antibiotic cocktail was administered for 3 days and then the animals were switched to regular drinking water for 2 days. A single dose of clindamycin (10 mg/kg) was administered intraperitoneally 1 day before *C. difficile* challenge. 16 h after infection (day 1), the mice were intragastrically administered with compounds suspended in 5% sodium carboxymethylcellulose (CMC-Na) solution. From the second to the fifth day, the mice were treated with drugs once daily. The normal group and model group were administered with solvent alone. Mouse weights and the development of disease symptoms were monitored daily. Animals that became moribund or lost >20% of their body weights were euthanized. All of the animals were euthanized at day 7. The mice were divided into the following groups (eight per group): Normal (5% CMC-Na solution), Model (5% CMC-Na solution), IBCL (50 mg/kg), and Vancomycin (50 mg/kg).

### Statistical Analysis

All of the experiments were repeated at least in duplicate independent experiments and the data are presented as mean ± standard error of the mean (SEM). Statistical significance between control and experimental values was calculated by one-way ANOVA, which was performed with Graph Pad prism 5 (GraphPad Software, San Diego, CA, United States). Significant values are represented by an asterisk: *p < 0.05, **p < 0.01.

## Results

### Antibacterial Activity of PFPE Against C. difficile

From Table 2 it can be seen that PFPE possess medium antibacterial effect against *C. difficile* ATCC 43255, BAA1803, and CICC 22951, and the MIC values were 8, 8, and 8 μg/ml, respectively. This result was consistent with the antidiarrheal description of PF in the Chinese Pharmacopoeia. Although PFPE MIC was no better than vancomycin and metronidazole, its antibacterial activity was outstanding compared to most natural products.

### Quantitative Analysis of the Major Constituents in PFPE and PF

**FIGURE 2:**
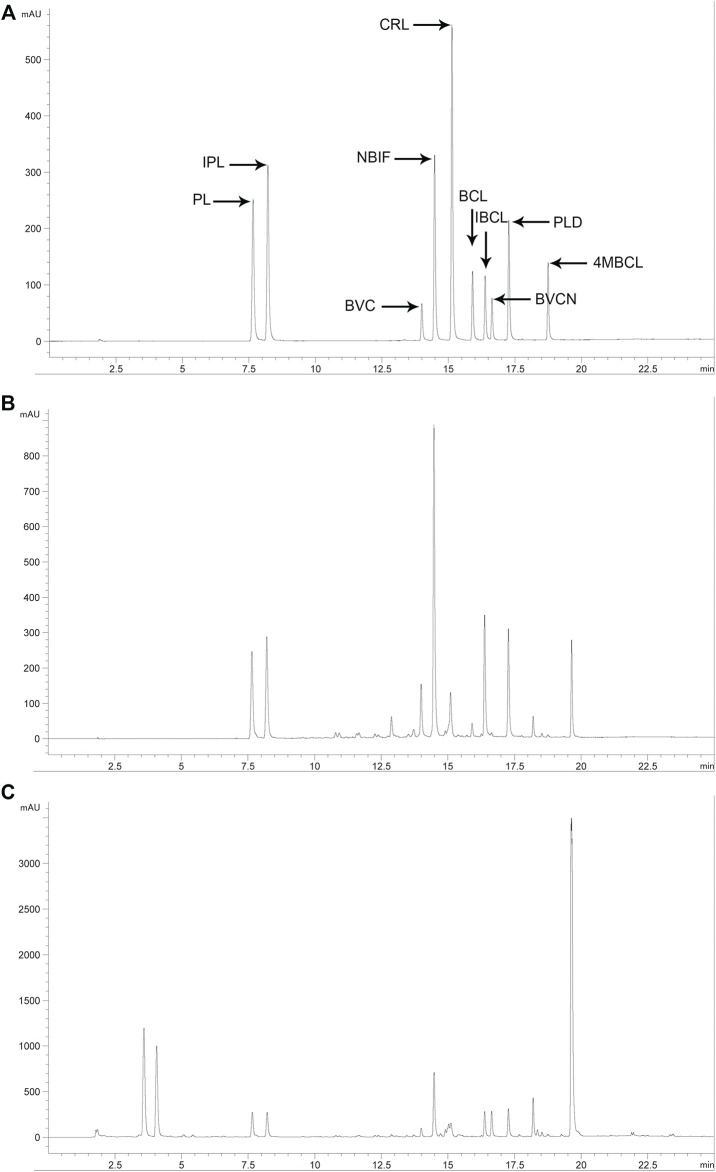
HPLC chromatogram of related standard compounds (**(A)** standard mixture at 50 μg/ml), PFPE (**(B)** 1 mg/ml), and PF (**(C)** 10 mg/ml) at 254 nm.

To identify the specific compounds in PFPE that are responsible for the significant antibacterial activity against *C. difficile*, the contents of PL, IPL, BVC, NBIF, BVCN, CRL, PLD, BCL, IBCL, and 4MBCL were measured. First, a reliable HPLC method was established with a suitable column and mobile phase. The major components in PFPE can be well separated by this method (Figure 2). Second, the standard curves of each compound (1.56–200 μg/ml) were established and *R* ^2^ was above 0.999 (Table 1). Finally, the contents of each component in PFPE and PF were well measured (Table 1). From the data, IBCL, NBIF, and BVC were the major compounds in PFPE with contents of 18.16, 13.80, and 13.38%, respectively. The contents of PL, IPL, CRL, BCL, and PLD in PFPE were medium, which ranged from 2 to 10%. The contents of BVCN and 4MBCL were 0.01 and 0.02%.

**TABLE 1:** Parameters for the quantitative analysis of the major components in PFPE and PF.

Compounds	Retention time (min)	Standard curve	*R* ^2^	Content in PFPE (%)	Content in PF (%)
PL	7.655	Y = 26.621x + 8.5681	1	5.12	0.28
IPL	8.214	Y = 32.311x + 9.8049	1	4.75	0.23
BVC	14.001	Y = 5.7026x + 0.3469	0.999	13.38	0.41
NBIF	14.481	Y = 28.028x - 7.7323	1	13.80	0.55
CRL	15.134	Y = 46.912x + 2.4243	1	1.97	0.15
BCL	15.909	Y = 9.9479x - 2.4117	1	2.31	—
IBCL	16.382	Y = 8.9919x - 3.11	1	18.16	0.64
BVCN	16.639	Y = 5.8599x - 0.6546	1	0.01	1.05
PLD	17.272	Y = 17.731x - 14.464	0.999	7.92	0.49
4MBCL	18.748	y = 10.135x - 2.1279	1	0.02	0.10
Total				67.44	3.90

—Not detected.

The contents of IBCL, NBIF, and BVC in PF were 0.64%, 0.55%, and 0.41%, which were also higher compared to other components, except with BVCN. In contrast from PFPE, the content of BVCN (1.05%) was highest in PF. The contents of PL, IPL, CRL, and 4MBCL in PF were 0.28%, 0.23%, 0.15%, and 0.15%, respectively. Therefore, the major phenolic constituents in PF except with BVCN were successfully extracted and transferred to PFPE. The contents of the phenolic compounds in both PF and PFPE were detected with the reliable HPLC method.

### Antibacterial Activity of the Major Constituents in PFPE Against C. difficile

To identify the antibacterial components in PFPE, antibacterial activities of relative compounds against *C. difficile* ATCC 43255, BAA-1803, and CICC 22951 were evaluated with the microdilution method. As summarized in Table 2, several compounds (PL, IPL CRL) were inactive (MIC >64 μg/ml) or partially active (BVC, NBIF, BCL, BVCN, PLD, MICs = 16–32 μg/ml) against *C. difficile*. IBCL and 4MBCL were highly active with MIC of 4 μg/ml to all three strains. Vancomycin and metronidazole were active to all the strains and the MIC ranged from 0.25 to 2 μg/ml.

**TABLE 2:** The MIC and MBC of test samples against *C. difficile*.

Compounds	Activity (μg/ml) against *C. difficile*					
	ATCC 43255		BAA-1803		CICC 22951	
	MIC	MBC	MIC	MBC	MIC	MBC
PFPE	8	16	8	16	8	8
PL	>64	—	>64	—	>64	—
IPL	>64	—	>64	—	>64	—
BVC	32	—	32	—	32	—
NBIF	16	—	32	—	32	—
CRL	64	—	64	—	64	—
BCL	16	—	8	16	8	16
IBCL	4	8	4	8	4	8
BVCN	16	—	16	—	16	—
PLD	16	—	32	—	32	—
4MBCL	4	8	4	8	4	8
VAN	1	8	2	8	0.5	8
MNZ	1	1	0.5	2	0.25	0.5

—Not tested.

The MBCs of the active compounds against *C. difficile* were also investigated. Both IBCL and 4MBCL showed significant bactericidal activities with MBC of 8 and 8 μg/ml. The MBC of PFPE was between 8–16 μg/ml to the different strains. The MBC of vancomycin was 8 μg/ml and was considerably high when compared to its MIC. For metronidazole, the MBCs were 0.5–2 μg/ml to the different *C. difficile* strains.

### Growth Curves for C. difficile

**FIGURE 3:**
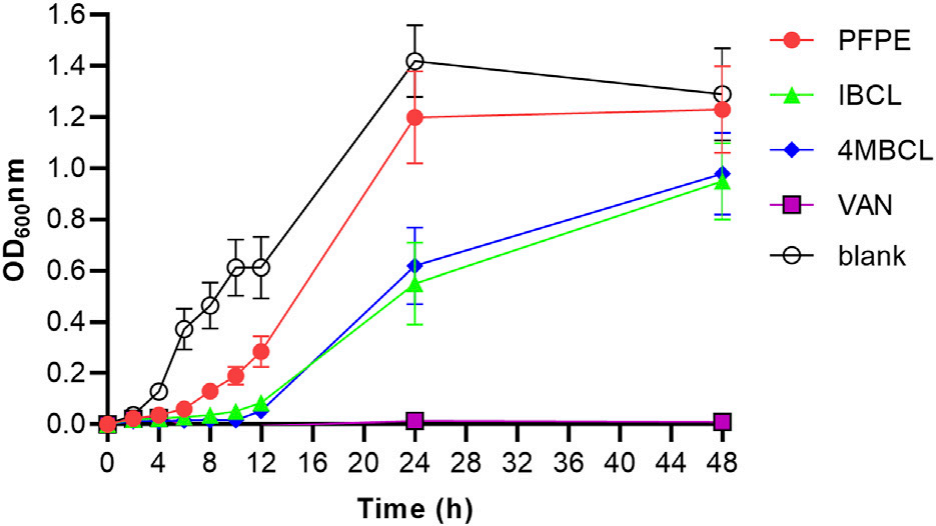
Growth curves of *C. difficile* with treatment of PFPE (0.5 × MIC, 4 μg/ml), IBCL (0.5 × MIC, 2 μg/ml), and 4MBCL (0.5 × MIC, 2 μg/ml). Vancomycin (1.0 × MIC, 1 μg/ml) was set as positive control and no-drug treatment group was set as blank.

The *C. difficile* growth curves obtained by optical method demonstrated different rates depending on the concentration of the samples used. The growth phase ended after 48 h. The level of inhibition increased with the increase of compounds concentration. PFPE, IBCL, and 4MBCL can partially inhibit the growth of *C. difficile* at a concentration of 0.5 × MIC. Compared to PFPE, IBCL, and 4MBCL showed more inhibition rates (Figure 3). The growth of *C. difficile* was totally inhibited by 1 × MIC vancomycin.

### Concentration-dependent Killing of C. difficile

**FIGURE 4:**
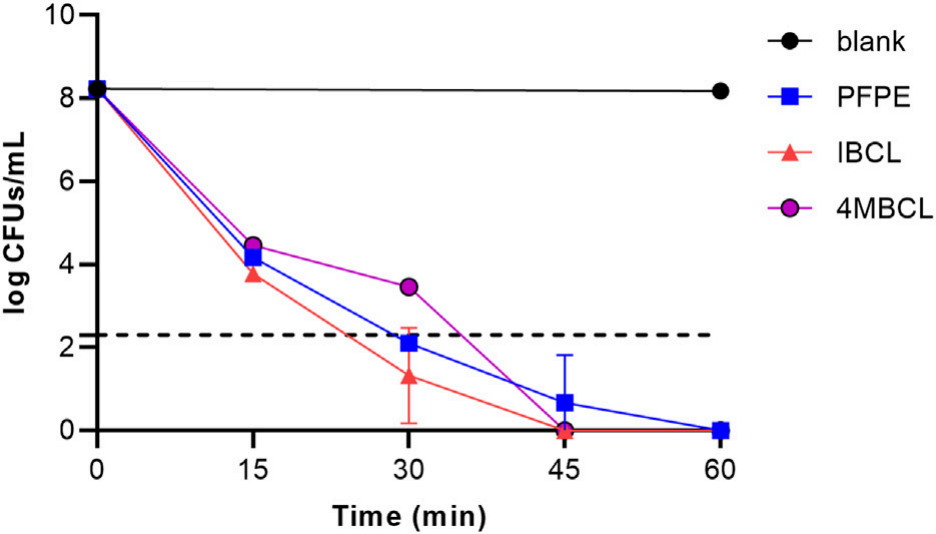
Killing kinetics of PFPE (4 × MIC, 32 μg/ml), IBCL (4 × MIC, 16 μg/ml), and 4MBCL (4 × MIC, 16 μg/ml) against stationary-phase *C. difficile*. A no-drug control was induced with each run. Samples of <200 CFU/ml were below the limitation of detection.

The bactericidal properties of PFPE, IBCL, and 4MBCL against *C. difficile* ATCC 43255 were evaluated. As shown in Figure 4, IBCL and 4MBCL effectively killed *C. difficile* at a concentration of 4×MIC. Accordingly, the killing activities of IBCL and 4MBCL were found to be rapid and concentration dependent. Within 15 min, the viability of stationary-phase bacteria was dramatically reduced by 4 logs after exposure to IBCL at 16 μg/ml. Moreover, 6 logs cells were killed at 30 min. Meanwhile, 4MBCL also rapidly reduced the viability of *C. difficile*, killing ≥3 logs in 15 min and about 4 logs in 30 min at 16 μg/ml. PFPE showed better bactericidal effect compared to 4MBCL at the concentration of 32 μg/ml (4 × MIC). PFPE killed about 4 logs bacteria in 15 min and reduced more than 6 logs cells in 30 min. At 45 min, residual cells of all treatments were under the limit of detection, which meant that more than 6 logs cells were killed after 45 min treatment. For normal control, the bacteria amounts were not significantly changed in 60 min (8 logs).

### Cell Morphology of C. difficile Seriously Influenced by PFPE, IBCL, and 4MBCL

**FIGURE 5:**
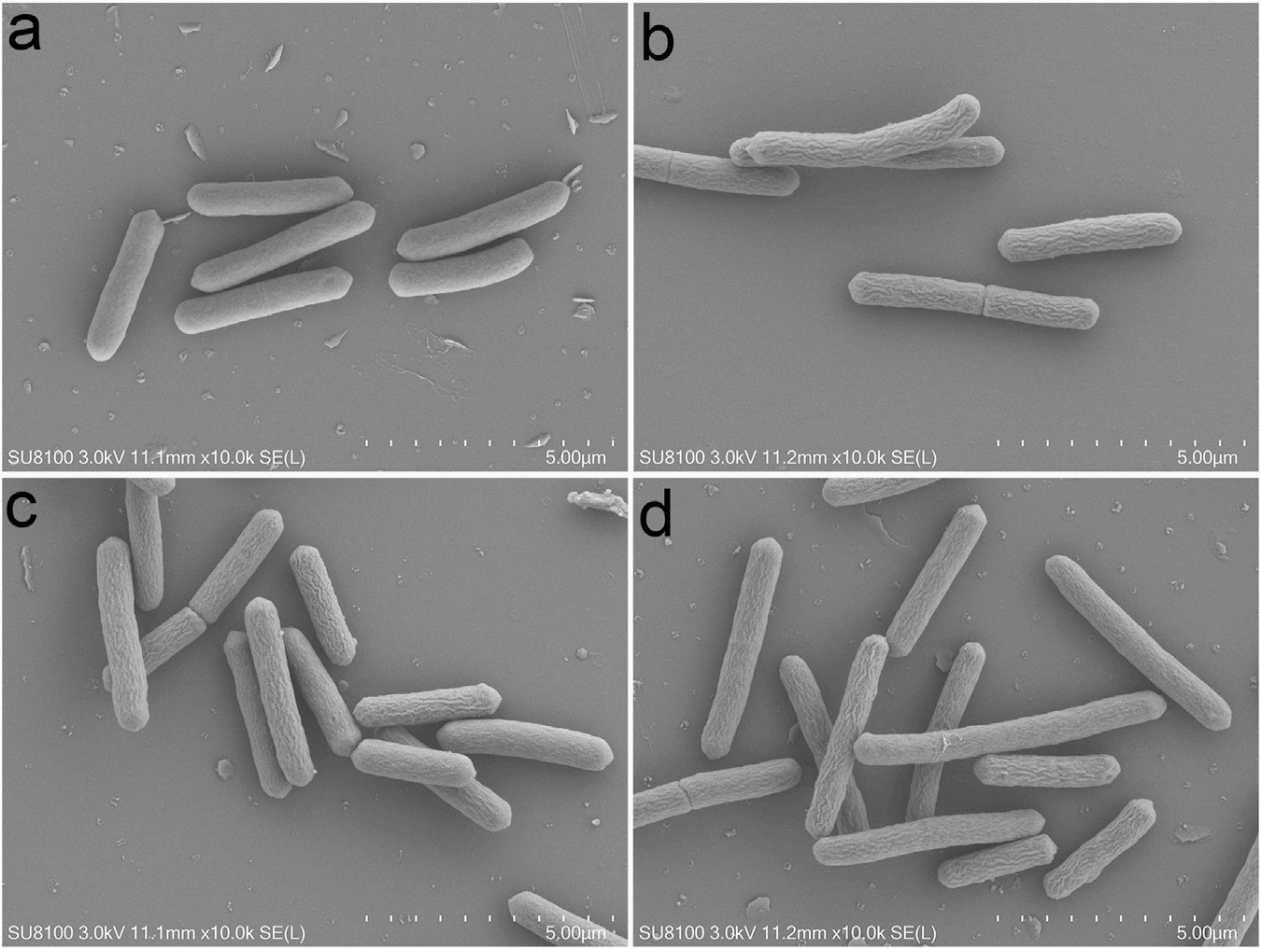
Morphology of *C. difficile* observed by SEM after drugs treatment for 2 h. **(A)** Blank. **(B)** PFPE at 16 μg/ml. **(C)** IBCL at 8 μg/ml. **(D)** 4MBCL at 8 μg/ml.

To investigate the influence of PFPE, IBCL, and 4MBCL on bacteria morphology, the SEM technology was applied. As shown in Figure 5, the morphology of *C. difficile* was greatly different after PFPE, IBCL, and 4MBCL treatment. After treatment by PFPE (16 μg/ml), IBCL (8 μg/ml), and 4MBCL (8 μg/ml) for only 2 h, the bacteria’s shape became wrinkle and less smooth. The results suggested that intracellular water loss occurred by damage to the bacteria’s membrane. The rapid change of cell morphology in 2 h was consistent with the rapid bactericidal activity.

### Cell Membrane Destroyed by IBCL

**FIGURE 6:**
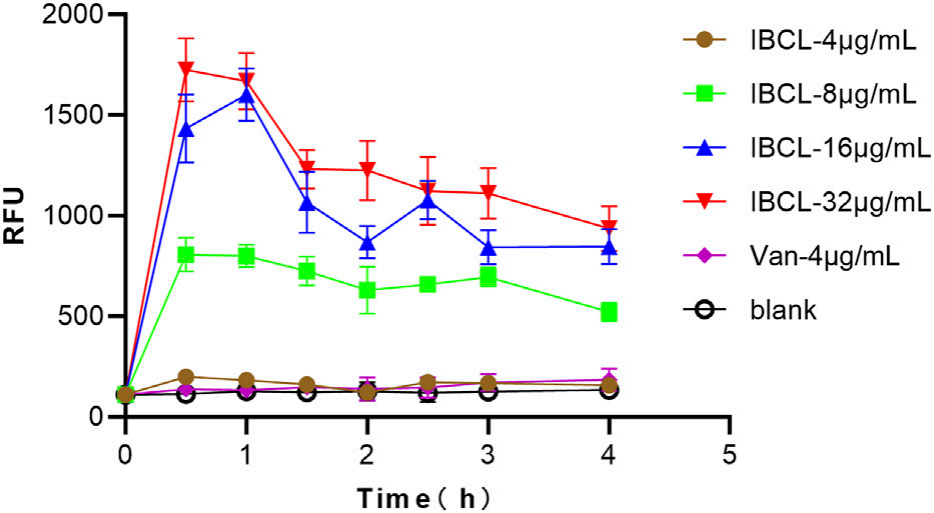
Fluorescence measurement of propidium iodide of *C. difficile* ATCC43255 bacteria treated with different concentration of IBCL and Vancomycin.

IBCL-treated *C. difficile* stained with PI after 0.5 h of exposure indicated damage in membrane integrity of *C. difficile* cells. The relative fluorescence unit (RFU) of PI was about 100–150 without any treatment, while the RFU was dramatically increased to about 1,500 after IBCL treatment. Moreover, the RFU of 16 μg/ml and 32 μg/ml IBCL treatments were higher than the 8 μg/ml IBCL treatment. In the 4 μg/ml treatment, the RFU was a little higher than no treatment. All of the results indicated that damage to the integrity of the cell membrane depended on the drug concentrations. However, the RFU with vancomycin treatment was almost unchanged, which was consistent to the mode of action of vacomycin. The RFU of IBCL treatments reached a peak at 0.5 h and then decreased slowly during the detection period by the rapid concentration of the bactericidal effect of IBCL (Figure 6).

### Cytotoxicity Study of PFPE and Active Contents

The cytotoxicities of PFPE, IBCL, and 4MBCL against human cancer cell line Caco-2 were evaluated with a CCK-8 cell viability kit. From Table 3, IBCL and 4MBCL showed moderate cytotoxicity with IC_50_ of 37.09 and 23.96 μg/ml, respectively. Therefore, when they are regard as drugs against CDI and used clinically, side effects should not be ignored due to their cytotoxicity. PFPE has low cytotoxicity and the IC_50_ was 271.3 μg/ml.

**TABLE 3:** Cytotoxicity of PFPE, IBCL, and 4MBCL on Caco-2 cell line.

Compounds	IC_50_(μg/ml)
PFPE	271.3
IBCL	37.09
4MBCL	23.96

### IBCL Protects Mice From C. difficile Infection

Mice preconditioned with antibiotics and challenged with *C. difficile* developed typical CDI symptom (weight loss, piloerection, hollow back, diarrhea, and less activity). To test the therapeutic efficacy of IBCL in preventing CDI on mice, IBCL was administered at 50 mg/kg dosage via oral gavage 16 h post infection and for five consecutive days.

**FIGURE 7:**
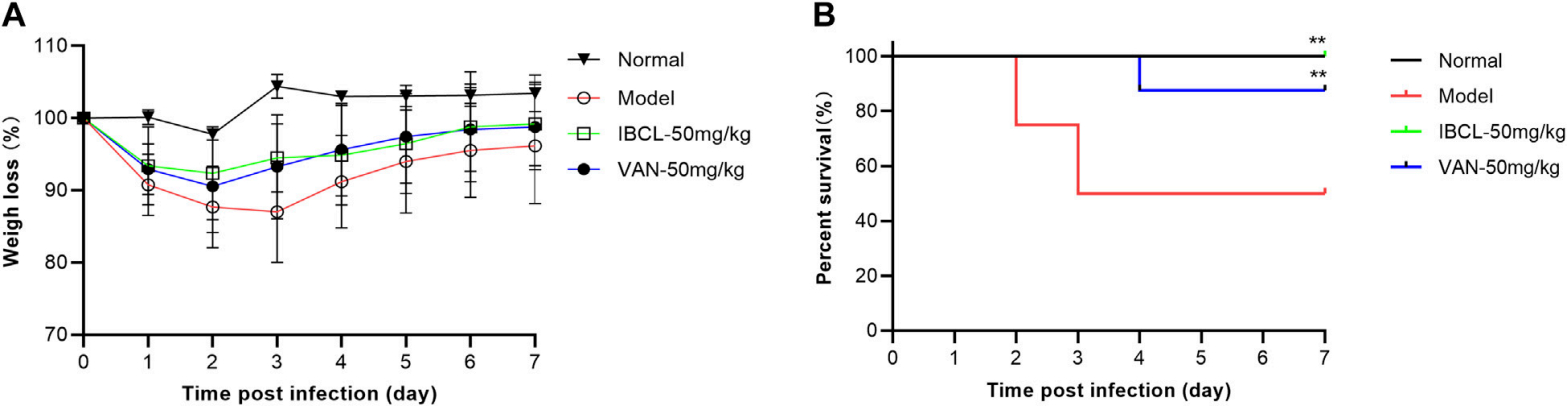
IBCL is protective *in vivo* on mice CDI model. **(A)** Weights loss of mice after challenge with *C. difficile* (10^5^ CFU/ml) on day 0 to day 7. **(B)** Mouse survival as determined by a log-rank/Mantel–Cox test. **p < 0.01 using one-way ANOVA. Data represent the eight mice/group.

From the second day, the mice of model group started to die and the survival rate was only 50% (Figure 7). For IBCL and positive drug vacomycin, the survival rates were 100 and 87.5%, respectively. The mice weights of the drug groups and model group were dramatically decreased after infection and began to recover from day 4. However, the body weights of IBCL group were higher than vancomycin and the model group. For CDI symptoms of piloerection, hollow back, diarrhea, and movement, IBCL and vancomycin groups were very slight when compared to the model group. These results suggest that IBCL can protect mice from CDI through the alleviation of clinical symptom, reduction of weight loss, and prevention of death. At the same dosage of 50 mg/kg, the therapeutic efficacy of IBCL may be better than vancomycin when considering weight loss and death rate.

## Discussion

CDI is an important infectious disease that is caused by *C. difficile* in humans, especially in hospital and other medical facilities. Vancomycin and metronidazole can effectively cure the disease thanks to their significant antibacterial activity. The high recurrent rate and constantly emerging drug resistance, which cause more expensive medical expense and treatment failure, make this issue more complicated. As the source of food and drugs, plants provide a wide range of products for human and animals. For a long time, scientists have searched drug candidates from natural products and have achieved success thanks to their safety and effectiveness.

As the seeds of *C. corylifolium*, PF is included in Chinese Pharmacopoeia. It has been proven to be antidiarrheal and was used for thousands of years in ancient China. Recently, studies have revealed that the flavonoids were the major secondary metabolites in PF, which may be the active constituents for its various activities (Zhang et al., 2016; Liu et al., 2022). In this study, we prepared the phenolic extract of PF and investigated the *in vitro* antibacterial activity against *C. difficile*. The significant antibacterial activity of PFPE prompted us to find the specific antibacterial compounds. Content determination showed that IBCL (18.16%), NBIF (13.80%), and BVC (13.38%) were the major components in PFPE. Correspondingly, the contents of IBCL, NBIF, and BVC in PF were 0.64%, 0.55%, and 0.41%, respectively. Additionally, the compound of retention time at 19.7 min might be bakuchiol. Bakuchiol is the major component of essential oil from PF (Adarsh Krishna et al., 2022). Although liposoluble components including bakuchiol were removed by in PFPE preparation, the content of bakuchiol was considerable high in PFPE. This study focused on search of active flavonoids against *C. difficile*. Thus, the precise content and antibacterial activity of bakuchiol were not investigated.

IBCL was active against Gram-positive *S. aureus* with MIC of 4 μg/ml to both MRSA and MSSA (Song et al., 2021). The MIC of IBCL for mycobacterial species was 64 μg/ml (Assis et al., 2022). Some natural and synthetic flavonoids have shown significant antibacterial activity against *C. difficile*, including Olympicin A (MIC = 1 μg/ml), xanthohumol (MIC = 15 μg/ml), and compound **189** (MIC = 2 μg/ml) (Wu et al., 2014; Cermak et al., 2017). In this study, the outstanding antibacterial activity of IBCL and 4MBCL against *C. difficile* was beneficial to the discovery of new agents for CDI therapy. IBCL, 4MBCL, and PFPE showed rapid killing effect against *C. difficile* in concentration-dependent manner and can significantly kill *C. difficile* about 4 logs in 15 min and ≥6 logs in 45 min. The killing effect of vacomycin needed a long time to be exhibited, which indicates that PFPE, IBCL, and 4MBCL had a different mode of action from vacomycin.

SEM detection and PI uptake results proved this prediction. Bacteria membrane of *C. difficile* was rapidly destroyed by IBCL, 4MBCL, and PFPE, leading to significant wrinkle in morphology. Further study found that the cell membrane integrity was quickly disrupted by various concentrations IBCL in 0.5 h, which may cause an increase of membrane permeability and loss of intracellular water, ATP, and protein. Finally, the leak of intracellular cell components leads to morphology change and cell death. Similar results of membrane destruction were observed when IBCL was applied on *S. aureus* and *B. subtilis* (Dzoyem et al., 2013; Assis et al., 2022). However, vacomycin, which inhibit bacterial cell wall glycoprotein synthesis, did not disrupt membrane integrity. The specific targets on membranes affected by IBCL are still not quite clear, and therefore, further studies of structure-activity relationship and mechanism are needed.

Recent cytotoxicity studies have shown that most flavonoids (IPL, BVC, BVCN, NBIF, IBCL, and BCL, etc.) in PF were medium toxic. The IC_50_ values were various to different cell lines and most of them ranged between 20 and 200 μg/ml (Song et al., 2018; Guo et al., 2021; Chen et al., 2022). In this study, the total content of IBCL and 4MBCL in PFPE was 18.18%, other compounds (49.26%) mentioned above may have low cytotoxicity to Caco-2 cells. Besides, the rest substance (32.56%) in PFPE that was undetectable by the HPLC method may not be toxic or stimulative on cell proliferation. These reasons lead to the low cytotoxicity of PFPE compared to IBCL. Natural products have been used for thousands of years in China due to the efficacy and safety. Overall, PFPE may be better for clinical use although the antibacterial activity was weaker than IBCL.

*In vivo* study revealed that IBCL can significantly alleviate the CDI symptoms and reduce the death rate in the mice model. The therapeutic efficacy of IBCL at 50 mg/kg dosage was better than vancomycin. The *in vitro* and *in vivo* results suggest that IBCL may be a promising lead compound to discover new molecules for CDI and further studies are needed.

## Conclusion

As the seeds of *C. corylifolium*, Psoraleae Fructus is described to be effective on diarrhea and enteritis in the Chinese Pharmacopoeia. The phenolic extract of PF and IBCL showed significant antibacterial activity against *C. difficile in vitro* with MIC of 8 and 4 μg/ml*.* IBCL can disrupt quickly bacteria membrane integrity in a concentration-dependent manner to achieve antibacterial action. IBCL effectively alleviated and reduced symptoms of weight loss and death in the mice model caused by CDI. Therefore, IBCL is a promising lead compound or drug candidate for CDI and should be further studied.

## Data Availability

The raw data supporting the conclusion of this article will be made available by the authors, without undue reservation.
